# MRI-Based Radiomics and Radiogenomics in the Management of Low-Grade Gliomas: Evaluating the Evidence for a Paradigm Shift

**DOI:** 10.3390/jcm10071411

**Published:** 2021-04-01

**Authors:** Ahmed Habib, Nicolina Jovanovich, Meagan Hoppe, Murat Ak, Priyadarshini Mamindla, Rivka R. Colen, Pascal O. Zinn

**Affiliations:** 1 Department of Neurosurgery, University of Pittsburgh Medical Center, Pittsburgh, PA 15232, USA; habiba@upmc.edu; 2 Hillman Cancer Center, University of Pittsburgh Medical Center, Pittsburgh, PA 15232, USA; jovanovich.nicolina@medstudent.pitt.edu (N.J.); meagan.hoppe@pitt.edu (M.H.); akm@upmc.edu (M.A.); mamindlap@upmc.edu (P.M.); colenrr@upmc.edu (R.R.C.); 3 Department of Diagnostic Radiology, University of Pittsburgh Medical Center, Pittsburgh, PA 15232, USA

**Keywords:** radiomics, radiogenomics, low-grade glioma, brain tumors, machine learning

## Abstract

Low-grade gliomas (LGGs) are tumors that affect mostly adults. These neoplasms are comprised mainly of oligodendrogliomas and diffuse astrocytomas. LGGs remain vexing to current management and therapeutic modalities although they exhibit more favorable survival rates compared with high-grade gliomas (HGGs). The specific genetic subtypes that these tumors exhibit result in variable clinical courses and the need to involve multidisciplinary teams of neurologists, epileptologists, neurooncologists and neurosurgeons. Currently, the diagnosis of an LGG pivots mainly around the preliminary radiological findings and the subsequent definitive surgical diagnosis (via surgical sampling). The introduction of radiomics as a high throughput quantitative imaging technique that allows for improved diagnostic, prognostic and predictive indices has created more interest for such techniques in cancer research and especially in neurooncology (MRI-based classification of LGGs, predicting Isocitrate dehydrogenase (*IDH*) and Telomerase reverse transcriptase (*TERT*) promoter mutations and predicting LGG associated seizures). Radiogenomics refers to the linkage of imaging findings with the tumor/tissue genomics. Numerous applications of radiomics and radiogenomics have been described in the clinical context and management of LGGs. In this review, we describe the recently published studies discussing the potential application of radiomics and radiogenomics in LGGs. We also highlight the potential pitfalls of the above-mentioned high throughput computerized techniques and, most excitingly, explore the use of machine learning artificial intelligence technologies as standalone and adjunct imaging tools en route to enhance a personalized MRI-based tumor diagnosis and management plan design.

## 1. Introduction

Low-grade gliomas (LGGs) are a group of heterogenous neuroepithelial malignant tumors of the central nervous system (CNS) that are classified by the World Health Organization (WHO) as II tumors. LGGs account for approximately 17–22% of all primary brain tumors (approximately 20,000 cases a year in the United States); they comprise of mainly astrocytomas and oligodendrogliomas [1,2,3]. LGGs are characterized by a variable molecular profile and subsequently a variable clinical course [4]. Numerous molecular markers are associated with LGGs; of those are *IDH* 1/2 mutations, which, when present with the *ATRX* and *TP53* loss, result in the diagnosis of a diffuse astrocytoma or anaplastic astrocytoma (grade II and grade III, respectively). On the other hand, if *IDH* mutations are found along with chromosomal *1p/19q* co-deletion then the diagnosis of oligodendroglioma or anaplastic oligodendroglioma is made [5]. The identification of these key molecular features in LGGs has led to further classification and better management schemes for LGG patients. *IDH* 1/2 mutations and the *1p/19q* co-deletion status are now considered to be an essential prognostic factor in LGG patients. In the clinical setting, these molecular markers are used to classify LGG patients post-surgically into high and low risk patients. Patients less than 40 years of age with a tumor size less than 4 cm with an *IDH* mutation and *1p/19q* co-deletion are considered low risk with a favorable prognosis overall and they are usually observed closely without the need of an immediate surgical intervention [6,7]. On the other hand, patients who are older with the same molecular profile but with a larger tumor size as well as being *IDH* wild type (WT) or *1p/19q* non-co-deleted are considered high risk and mandate an immediate intervention [7].

There is currently no universal treatment paradigm for LGGs due to the clinical profile variability, the effect of demographic factors (age, tumor location, etc.) and the tumoral molecular landscape of each patient tumor [8,9]. Nevertheless, current LGG management consists of a surgical resection followed by chemotherapy and radiation therapy for high risk groups [10]. The introduction of non-invasive modalities such as MRI-based radiomic and radiogenomic imaging has demonstrated a potential tool that could benefit the diagnostic process, follow-up and, more recently, predict the tumor response to therapy in neurooncology [11,12].

**Figure 1:**
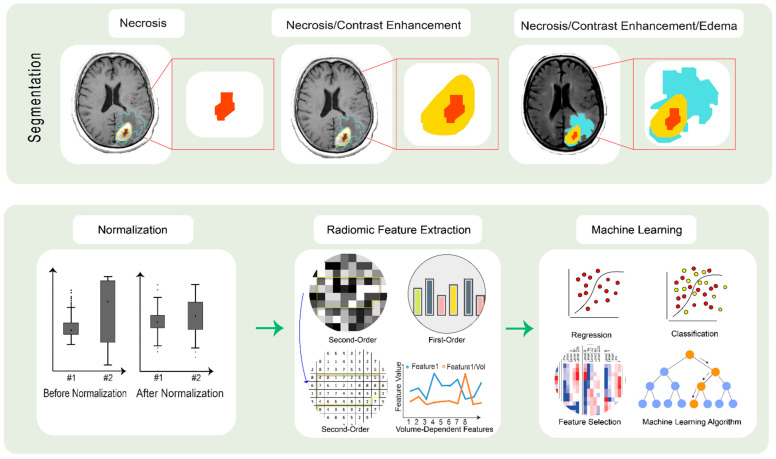
Radiomics pipeline for brain tumors. Top line: segmentation of the three imaging phenotypes: necrosis (**left**), enhancement/edema (**middle**) and enhancement/edema/invasion (**right**). Bottom line: radiomics feature extraction from MR images, data value normalization and volume-dependent feature generation are followed by predictive modeling for outcomes.

Radiomics is an emerging translational field in imaging; it refers to the high throughput feature extraction method that uncovers microscale quantitative information within conventional and advanced imaging modalities. Radiogenomics is the association and prediction of imaging features with the genomic composition of the tumor [13]. These novel techniques can be used to better understand the genomic basis of cancer and how it relates to high resolution imaging [14]. In particular, studies have focused on tumor spatial heterogeneity [15,16], treatment response [15,16], molecular classifications [17] and tumor microenvironment immune infiltration [16] (Figure 1). Radiomics and radiogenomics have also been used to predict histological features, grade or even overall survival (OS) in LGGs. Radiomics is ideal for a cost-efficient clinical translation as a complementary tool as it is non-invasive and characterizes the entire three-dimensional tumor landscape inclusive of the spatial heterogeneity [18]; furthermore, imaging features are extracted from scans routinely obtained from patients as part of the standard of care. Nevertheless, cumulative literature discussing the role of radiomics and radiogenomics in LGGs is still scarce. In this article, we review recently published studies discussing the potential role and applications of radiomics and radiogenomics in the diagnosis and prognosis of LGGs. We also explore the possibility of using such technologies as standalone or adjunct tools to improve care for LGG patients.

## 2. Current Insights into the Diagnosis and Management of LGGs

The current diagnostic process of an LGG revolves around conventional magnetic resonance imaging (MRI) or computed tomography (CT) imaging plus a histopathological diagnosis. On CT imaging, LGGs appear as an ill-defined area of low attenuation. Although the use of CT imaging could help direct the clinical decision, it is inferior to MRI, which is the gold standard diagnostic imaging modality for LGGs and brain tumors in general. On MRI, LGGs appear as homogenous low signal diffuse lesions on T1-weight, high intensity on T2 and Fluid-Attenuated Inversion Recovery (FLAIR) images. Calcifications may also be present as T2 hyperintensity or T1 hypointensity in up to 20% of LGGs. Although contrast enhancement is mainly a characteristic feature that is associated with high-grade gliomas (HGGs), LGGs can also rarely demonstrate contrast enhancement to a lesser extent [19].

The medical management of LGG patients depends on multiple factors (age, tumor size and molecular profile). The current surgical management of LGGs revolves around achieving a maximal safe resection followed by chemotherapy and or radiation therapy [6,20,21]. Currently, a stepwise diagnostic algorism is implemented for each LGG patient post-surgically starting with determining demographic factors; age >/< 40 years and tumor size >/< 4 cm followed by determining the molecular profile for each patient with immunohistochemistry to define the *IDH* 1 (which represent 90% of all *IDH* mutations) and the *1p/19q* co-deletion status [6,7]. Using this diagnostic algorithm, patients can be classified into two groups; high risk vs. low risk [7]. Despite the lack of multicenter controlled trials that assess the beneficial role of the extensive resection of LGGs, Smith, et al. [22] retrospectively analyzed 216 patients with LGGs surgically treated at the University of California San Francisco (UCSF) between 1989 and 2005. They found that the extent of the resection was a significant predictor associated with OS and progression-free survival (PFS). The five years’ survival rate in patients with a 90% resection or more was 97% whereas it was 76% in patients with less than a 90% extent of resection. With respect to chemotherapy, there is no universal consensus on when to administer chemotherapy in LGGs; it could be administered as neoadjuvant or adjuvant and also it can be administered concomitant with radiotherapy [8]. In the recently published CODEL: Phase III trial results in which the role of radiotherapy only vs. chemoradiation vs. chemotherapy only in newly-diagnosed *1p/19q* co-deleted WHO grade III oligodendroglioma in adult patients was investigated, they found that patients who received chemotherapy only (temozolamide) had the shortest progression-free survival compared with patients who received radiation therapy [23].

In the 2016 RTOG9802 trial (NCT00003375), observation vs. radiation therapy with and without chemotherapy for patients with LGGs were investigated and they found that the surgical resection had a superior survival advantage over both chemotherapy and radiation therapy combined [24].

## 3. Implications of Radiomics in an LGG Diagnosis

MRI-based radiomics is a non-invasive modality that has become a special area of interest in the grading of tumors. Traditionally, a stereotactic-guided needle biopsy (SNB) along with a standard surgical resection are the gold standard methods of tumor diagnosis and grading followed by a subsequent histopathological and genomic diagnosis [25,26,27]. However, an SNB is an invasive clinical procedure and thus runs the risk of surgical complications (e.g., infection, intracerebral hemorrhage) [25,26,28]. Additionally, samples collected through an SNB are not necessarily an accurate representation of the whole tumor landscape as gliomas are known to be heterogeneous and the classical SNB may not always reflect the phenotype of the entire tumor or perhaps miss important genomic aberrations in the lesion [29,30]. This has led to ample research on non-invasive methods such as radiomics and radiogenomics that have the ability to distinguish HGGs from LGGs as well as potentially predict different LGG subtypes (astrocytomas, oligodendrogliomas, etc.). In addition, radiomics and radiogenomics are being tested as a means for guiding an SNB to the site of the highest yield of diagnosis [31,32].

Determining the type of glioma is imperative for an early treatment plan implementation and an assessment of patient prognosis. Recently, Cho et al. performed a multimodal analysis of 285 patients with brain tumors using T1-weighted, T1-contrast enhanced, T2-weighted and FLAIR MRI [33]. They assessed three regions of interest (ROIs) using a minimal redundancy maximum relevance (MRMR) algorithm and narrowed down the image features to the most stable and relevant in classifying the glioma grade (spherical disproportion, contrast, compactness and autocorrelation). Using these features in three classifier models (logistics, support vector machines and random forest classifiers) through a five-fold cross validation method for separating patients into training and test data yielded an average area under the curve (AUC) of 0.9030 for the test cohort and an average of 88% accuracy, 95% sensitivity and 70% specificity for distinguishing LGGs from HGGs [33]. Su et al. had similar results when they employed a multicontrast radiomics model with volume, compactness 1, compactness 2, spherical disproportion, sphericity and surface 2 volume features to differentiate HGGs from LGGs (AUC = 0.911, sensitivity: 85%, specificity: 85%) [34]. They also built a multicontrast model that identified radiomics features indicative of Ki-67 labeling index induced tumor proliferation; this yielded a predictive performance of 80% sensitivity and 87% specificity (AUC = 0.936) [34]. Other studies have used the featural patterns of radiomics to map different markers such as S-100, vimentin and CD34 expression patterns (specificities of 91%, 72% and 88%, respectively) and have further confirmed that glioma grading, as well as metastasis, can be accurately determined using radiomics [35,36,37]. These data suggest that radiomics could become a robust, non-invasive tool for glioma grading as well as used to further individualized medicine by shaping each patient treatment plan around patient-specific radiomics patterns.

## 4. MRI-Based Radiomics Could Predict Molecular Markers and Overall Survival in Diffuse Lower Grade Gliomas

The ability to distinguish diffuse LGGs subtypes is essential for implementing an effective treatment plan and consequently maximizing progression-free survival. Identifying *IDH* 1/2 and the *1p/19q* loss of heterozygosity status and high vs. low risk group determination are fundamental to modern LGG classification and prognostications [38]. In previous studies, it has been shown that *IDH-WT* LGGs have more post-contrast enhancement than *IDH*-mutant gliomas [39,40]. Liu et al. went on to further investigate these differences by building a radiomics model using T2-weighted images and logistic regression analyses to solidify radiomic features that predicted an *IDH*-mutant-specific signature. They were able to achieve an AUC of 0.86 with only 10 features and were able to predict 100% of *IDH*-mutant gliomas from *IDH*-WT gliomas using 86 features (AUC = 1.0) on the training set with 158 patients and an AUC of 0.99 using 86 features on the validation set with 102 patients [13]. The ability of radiomics to make this distinction was further confirmed by Arita et al. with the accuracy of delineation for their radiomics model being 82% and 83% for the training and validation sets, respectively. The implementation of MRI structural atlas location data improved these accuracies to 85% and 87%; this suggests that the inclusion of tumor location data could further improve the brain tumor grade and subtype differentiation abilities of radiomics models [41].

Radiomics has been shown to aid in distinguishing between *IDH*-mutant co-deleted *1p/19q* tumors (oligodendrogliomas) and *IDH*-mutant non-co-deleted *1p/19q* tumors (astrocytomas). Patel et al. showed that a complete (or near-complete) hyperintense signal on a T2-weighted MRI in combination with a hypointense signal on a FLAIR, except for a potential hyperintense peripheral rim, was 100% predictive of *IDH*-mutant astrocytomas [42]. This radiological phenomenon has been termed a T2-FLAIR mismatch. Broen et al. further confirmed this specificity of the T2-FLAIR mismatch for diffuse and anaplastic astrocytomas by completing a retrospective study of diffuse astrocytoma (*IDH*-mutant), diffuse oligodendroglioma (*IDH*-mutant *1p/19q* co-deleted), anaplastic astrocytoma (*IDH*-mutant), anaplastic oligodendroglioma (*IDH*-mutant *1p/19q* co-deleted) and *IDH*-*WT* (Glioblastoma-like) T2-FLAIR images [43]. Reviewers scored the T2-FLAIR mismatch sign as being present in 34 of 70 diffuse astrocytoma tumors, four of five anaplastic astrocytoma tumors and 0 of 79 diffuse oligodendroglioma, anaplastic oligodendroglioma and *IDH*-WT tumors, making the specificity of this study 100% for differentiating astrocytomas from other LGGs [43]. Additional studies have yielded a similar specificity of T2-FLAIR mismatch for astrocytomas and have even correctly identified the *1p/19q* co-deletion status in patients whose biopsy histology findings were not suggestive of astrocytoma [44,45].

The use of radiomics in differentiating astrocytomas from oligodendrogliomas has also been studied via a textural imaging radiomics model conducted by Zhou et al. [38]. In their study, they used a multivariate analysis of grey level run-length matrix (GLRLM) low gray level run emphasis (LGRE; T1-CE), gray level size zone (GLSZM) short zone low gray level emphasis (SZGHE; T2-W) and GLRLM long run high gray level emphasis (T2-weighted) features to determine the *1p/19q* deletion status of various tumors. Their analysis was carried out using training and testing of the samples through a bootstrapping method (n = 100 rounds) and yielded an AUC of 0.96, a sensitivity of 90% and a specificity of 89% [38]. The high sensitivity and specificity of radiomics models for identifying astrocytomas could make it an imperative diagnostic tool in clinical neurosurgical cases and, therefore, being able to diagnose these tumors early in the progression via radiomic patterns and being able to administer chemotherapeutics early in the tumor progression could significantly improve both the progression-free survival (PFS) and disease-specific survival of patients [12,46,47,48].

In addition to the T2-FLAIR mismatch, the hypermethylation of the O-(6)-methylguanine-DNA-methyltransferase (*MGMT*) promoter is a known prognostic biomarker for astrocytomas. Wei et al. used a combined radiomics model consisting of a CE T1-weighted, a T2-weighted FLAIR and an apparent diffusion coefficient (ADC) to find a fusion radiomics signature specific to the *MGMT* promoter methylation [49]. Their model was able to differentiate between *MGMT* methylated tumors and *MGMT* non-methylated tumors with an AUC of 0.925 in the training cohort and 0.902 in the validation cohort. Differentiating *MGMT* methylated astrocytomas early on is especially important for identifying tumor susceptibility to temozolomide chemotherapy treatment as well as predicting the efficacy of Carmustine wafer implantation [49,50].

Additionally, radiomic models have been successful in the survival prediction of LGGs [51,52]. In a cohort of 296 LGG patients, Choi et al. showed that an analysis of 71 texture features from preoperative MRI scans through training (n = 205) and testing (n = 91) could predict the overall survival (OS) with an AUC of 0.62 on the testing set. A combined model generated by the integration of clinical variables into the radiomics model showed a significantly improved OS prediction (AUC = 0.70).

In a computed tomography study by Salto et al., the presence of calcification was significantly correlated with the *1p/19q* loss of heterozygosity (LOH) (*p*-value = 0.0001; positive predictive value (PPV) = 91%); all 78 patients with calcified tumors were diagnosed to have oligodendrogliomas [53]. Radiomics studies analyzing the specificity and sensitivity of calcification as a predictor of oligodendrogliomas are encouraged to elucidate whether this can be a reliable method of tumor classification. Furthermore, moving on from qualitative analyses, such calcification present or not in actual whole tumor complex radiomic MRI assessments will help to refine those predictive models in a quantitative fashion.

## 5. Predicting Clinical Outcomes in Patients with an LGG

Liu et al. investigated whether or not radiomics-based analyses could successfully predict epilepsy in patients with an LGG [54]. In this retrospective study, the authors enrolled 286 patients that were histopathologically diagnosed with grade II LGGs and classified them into two main cohorts; a primary (n = 194) and a validation (n = 92) cohort. Patients were surgically treated at Beijing Tiantan Hospital between September 2008 and March 2015 and had no history of craniotomy or a stereotactic biopsy. The primary and validation cohorts stratified patients into epilepsy (n = 136; n = 60) and no epilepsy (n = 58; n = 32) groups [54]. A total of 475 quantitative imaging features were extracted, falling into three categories: location features using a coordinate system (7), three-dimensional imaging features (349) and statistically significant (*p* < 0.05) interaction features between location features and imaging features (119). The program Elastic net (E-net) picked the 11 most predictive features and the radiomics signature was determined with a linear combination. The radiomics signature had a classification accuracy of 79.38% and an AUC = 0.8754 in the primary cohort and classification accuracy of 75% and an AUC = 0.8162 in the validation cohort, demonstrating that a radiomics analysis could be effective in predicting LGG-related epilepsy. The radiomics signature was combined with the clinical characteristics of sex, age and histopathology in a nomogram that predicted the probability of epilepsy. The C-index of the nomogram was 0.8769 (95% CI: 0.8303–0.9235) within the primary cohort and 0.8152 (95% CI: 0.7311–0.8993) within the validation cohort, suggesting it could be a useful clinical tool for rapid epilepsy prediction. The study overall demonstrated that a radiomics analysis could be useful for the individualized evaluation, prediction and management of LGG-related seizure events.

## 6. Radiomics as a Prognostic Tool in an LGG

MRI-based radiomics features have been shown to be a reliable tool for distinguishing HGGs from LGGs as well as LGG subtypes; researchers have also investigated the value of radiomics as a prognostic tool in patients with diffuse LGGs. In their study, Qian et al. identified six radiomics features of LGG MRI images: Autocorrelation, High Gray Level Run Emphasis (HGLRE), Short Run High Gray Level Emphasis (SRHGLE), Average, SumVariance and Variance [40]. Furthermore, they calculated the radiomics features that were significantly correlated with OS (*p*-value < 0.05). From these data, they were able to calculate a radiomics risk score to stratify LGGs into high and low risk groups. A multivariate Cox analysis confirmed that this risk score could stand as an independent prognostic factor (*p*-value = 0.042) [40].

Liu et al. similarly tested the prognostic capability of *IDH*-specific radiomics features by extracting a compact radiomics signature with features that had a *p*-value < 0.05 after a univariate Cox regression [55]. They then used each feature to create a risk evaluation that was able to stratify the LGG patients into high risk and low risk groups as well as stratify the *IDH*-mutant subtype further into high risk and low risk groups. Notably, the OS for the *IDH*-WT high risk group and the *IDH*-mutant high risk group was not significantly different (*p*-value = 0.199) [55].

Both of these studies performed radiogenomics analyses to look at the transcriptome differences between the high risk and low risk LGG groups. Genes such as *ERCC1*, *G6PD*, *SOX9* and *EGLN2*, which are involved in cell growth and metabolic processes, as well as genes associated with hypoxia, angiogenesis and stem cell proliferative oncogenic functions were significantly enriched in the high risk groups. These transcriptome differences could explain the increased aggressiveness of the tumors denoted high risk by radiomic patterns [40,49]. These studies, as well as data from other studies, show that radiomic analyses have the potential to be a powerful prognostic tool that predicts the progression of LGGs based on distinct radiogenomic patterns [38,56,57].

## 7. Pushing the Envelope and Future Directions for Radiomics in Neurooncology

Improvements in radiomics techniques continue to be made. A new form of radiomics called deep learning-based radiomics (DLR) has been developed in recent years [58,59]. In DLR, deep neural networks are trained to recognize certain featural patterns, normalize the image information for an accurate segmentation and directly extract high throughput image features. This may overcome the need to manually delineate image segmentation and the lack of standardized image feature extraction seen in traditional radiomics [60,61]. Li et al. delineated the *IDH1* status in LGGs using both traditional radiomics and DLR to see if these two models distinguished tumors with different accuracies. After extracting high throughput features from the convolutional neural network (CNN) of the DLR, they used statistical analyses to find a distinct radiomics signature that differentiated the *IDH1* mutation status. This resulted in an AUC of 92% when one imaging modality was used (T2) and an AUC of 85% when multiple-modality MR images were used; both of these DLR methods were better at distinguishing an *IDH1* mutation than traditional radiomics (AUC = 0.86) [61]. Diffusion tensor imaging (DTI) radiomics has also been shown to predict the *IDH* mutation status of LGGs (AUC = 0.9) greater than conventional radiomics (AUC = 0.835) [62].

## 8. Conclusions

**Figure 2:**
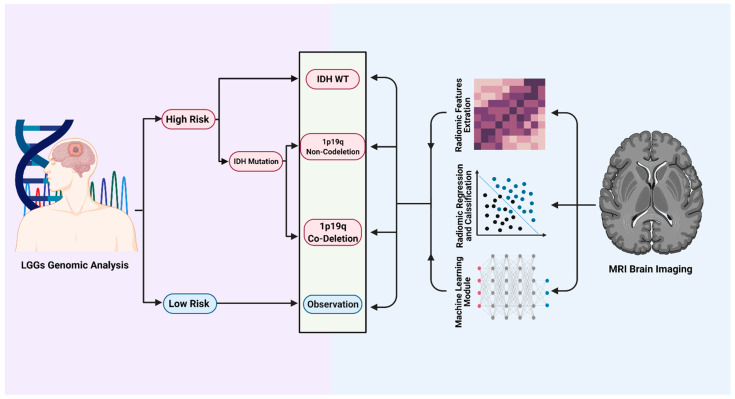
Using radiomics as a tool to predict the molecular landscape of LGGs. A graphic representation showing how radiomics and machine learning can be incorporated into the clinical setting to predict and classify patients with low-grade gliomas into high risk vs. low risk groups based on *IDH* and *1p19q* co-deletion status. The current data available support radiomics as a robust reliable tool that can non-invasively classify those patients by extracting radiomics features from MRI brain scans. Created with BioRender.com.

An LGG compared with an HGG portends a more benign clinical course and better survival rates; it is well established that the early diagnosis and gross total resection of an LGG can achieve long-term remission in low risk patient groups [61,62]. The synergy of multidisciplinary teams (neurosurgery, neurology, epileptology, neurooncology and neuroradiology) in LGG patient management is paramount particularly with the addition of high dimensional quantitative datasets such as deep learning-based radiomics. Computer data scientists/analysts, bioinformaticians and statisticians are becoming increasingly important as this field is undergoing a disruptive transformation. The introduction of MRI-based radiomics and radiogenomics analyses represents a non-invasive and cost-efficient adjunct tool that can extract quantitative information to augment clinical decision making (Figure 2). It is certain that once MRI-based non-invasive techniques are advanced enough, the need for surgical tissue diagnosis through biopsy may be reserved only for a subset of MRI non-diagnostic cases. Current data suggest that radiomics-derived features and patterns are capable of reliably classifying as well as predicting the PFS and OS of patients with diffuse LGGs. The continued refinement of radiomic models as technology evolves and large multicenter trials are needed to corroborate the above-mentioned studies and advance the LGG diagnosis, treatment and follow-up paradigm for our patients.
